# Influence of Chemical Conditions on the Nanoporous Structure of Silicate Aerogels

**DOI:** 10.3390/ma3010704

**Published:** 2010-01-26

**Authors:** Katalin Sinkó

**Affiliations:** Institute of Chemistry, University of Loránd Eötvös, Budapest, H-1117, Hungary; E-Mails: sinko@chem.elte.hu; Tel.: +36-1-372 2501

**Keywords:** nanoporous structure, silica aerogel, silicate aerogel, chemical influence

## Abstract

Silica or various silicate aerogels can be characterized by highly porous, open cell, low density structures. The synthesis parameters influence the three-dimensional porous structures by modifying the kinetics and mechanism of hydrolysis and condensation processes. Numerous investigations have shown that the structure of porous materials can be tailored by variations in synthesis conditions (e.g., the type of precursors, catalyst, and surfactants; the ratio of water/precursor; the concentrations; the medium pH; and the solvent). The objectives of this review are to summarize and elucidate the effects of chemical conditions on the nanoporous structure of sol-gel derived silicate aerogels.

## 1. Introduction

This review is largely devoted to structural studies of nanoporous silica and various silicate aerogels. Aerogels are highly porous, open cell, low density foams. The aerogels are sol-gel derived nanostructured materials with many favourable properties owing to their high porosity. Aerogels are considered as an entirely new class of solid state nanostructural porous materials. Their structures are typically built-up by nano-sized particles, which form an inorganic framework and the majority of the pores also fall in the nanoscale range. As a result of this unique nanostructure, these ultralow-density materials exhibit many interesting and unusual properties. Since the potential applications of aerogels depend mostly on their nanostructure, e.g., the pore size distribution, a study of the pore morphology is of primary importance.

Silica aerogels are attractive candidates for many unique thermal, optical, catalytic, and chemical applications due to their low density, high mesoporosity, small refractive index, and low thermal conductivity [1,2,3,4,5,6,7,8,9,10,11,12,13,14,15,16]. Silicate aerogels are typically mesoporous materials. The silicate gel networks are often described as having fractal geometry architecture but they may be randomly packed colloidal aggregate systems, too [17]. The structure of silica aerogels can be generally characterized by high surface area ~1,000 m^2^ g^-1^ [18], low density ~ 50 g cm^-3^ [19], high optical transmission ~90% [20], high porosity ~ 99% [21], low thermal conductivity ~ 0.05 W/mK [22] and low dielectric constant ~ 2 [23]. Specific surface areas up to 1,400 m^2^ g^-1^ [1,2,3,23,24,25], bulk densities of 20–240 g dm^-3^ [1,2,3,23,24,25] and heat conductivities below 0.02–0.08 W/mK [10,24] have been published. The approximate values of the pore size are between 5 and 100 nm [23,24,25], with an average pore diameter between 20 and 40 nm [23,24,25]. These structures are composed of small spherical silica clusters 3–4—50–100 nm in diameter connected to each other and forming three-dimensional networks. The silica aerogels prepared by supercritical drying are always amorphous.

The knowledge and the control of porous structures of aerogels are very important from the viewpoint of their applications in the field of separation and catalysis techniques. The usual route for aerogel preparation is the sol-gel technique. The sol-gel process allows an excellent control of the microstructure of silica and silicate gels from the earliest stages of procedure. The structure of aerogels can be mostly tailored by solution chemistry of the sol-gel technique. Numerous investigations have published numerous variations of the synthesis conditions (e.g., the type of precursor, solvent, and catalyst; the ratio of H_2_O/Si-precursor; the initial concentration of precursors, temperature, and pressure) which cause modifications in the structure. This review focuses on a summary of the reported chemical influences on the nanoporous structure of silica and silicate aerogels. The effects of initial materials; pH; catalyst; precursor concentration; water content; solvent; modifying agents; polymer; and incorporation of metal ions are detailed in this paper. Only a brief description of the influence of chemical agents on the aerogel structures will be presented here considering the very wide range of modifying agents. Special attention is given to the effect of metal ions embedded in the silica matrix. This field has been less emphasized in the reported monographs.

## 2. Sol-Gel Synthesis

The object of the sol-gel procedure is to prepare gel monoliths. One of the sol-gel routes synthesizes discrete colloid particles, *i.e.,* sols, as first step [24]. The sol must be condensed to gel in a time-consuming step. In another route, the interconnected gel network can be directly produced from the initial material mixtures [24]. A distinction can be made between several sol-gel processes even by the catalysis. In the “one step” route, the silica precursor is subjected to mixing with either an acid or base catalyst in a solvent. The catalysis favours the hydrolysis and condensation reactions leading to a gel formation [25]. In the “two step” acid-base sol-gel method, an acid-catalyzed prepolymerization of the silica precursor is the first step, and the second is a condensation reaction under alkaline conditions.

The usual precursors for aerogel preparation may be organic alkoxides, acetates, and inorganic salts, such as nitrates or chlorides. The most common silica precursors are the silicon alkoxides (tetramethoxysilane, TMOS; tetraethoxysilane, TEOS) and aqueous solution of sodium silicate (water glass) [1,2,3,24,25]. The tetraalkoxysilanes react very slowly in alcoholic and aqueous solutions, therefore, acid or base catalysis is necessary. The catalysts introduced in the polycondensation stage of preparation may be acids (e.g., hydrochloric, nitric, oxalic, and acetic acids) or bases (e.g., ammonia, sodium hydroxide). The alkoxysilanes are insoluble in water, thus a mutual solvent must be normally used as a homogenizing agent. Among the classes of solvents, alcohols are largely used, but other polar (H_2_O, acetone) and non-polar solvents (tetrahydrofuran, dioxane, benzene) may also be used [1,2,3,24,25].

A typical processing of silicate gels is the polycondensation of monomeric tetrafunctional alkoxide precursors ⌠Si(OR)_4_⌡ in the presence of mineral acid (e.g., HCl) or base (e.g., NH_3_). The essential reactions performed during the gelation are the hydrolysis and condensation processes of the precursors. The hydrolysis reaction replaces alkoxide groups (OR) with hydroxyl groups (OH). Subsequent condensation reactions involving the silanol groups produce siloxane bonds (Si–O–Si) and alcohol (ROH) or water. During the gelation, the number of siloxane bonds grows and consequently the number of silanols (Si-OH) and alkoxide groups reduces.

**Figure 1:**
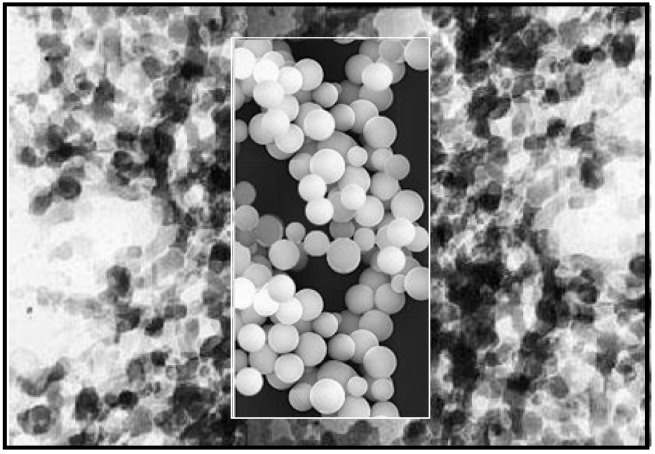
TEM image of silica aerogel (×200,000).

A super- or subcritical drying must follow the gelation in order to remove the pore liquid and obtain aerogels. In supercritical drying, the liquid can be removed from the pores in supercritical state. There is no liquid-vapor interface and no capillary pressure. Thus the supercritical drying process can avoid the drying shrinkage [5,14,24,25,26]. In the past 20 years, the use of supercritical carbon dioxide as a solvent for drying of gels containing organic solvent has made the process safer and more economical [27,28]. Figure 1 represents the nanostructure of the silica aerogels.

A lot of research to avoid the high cost of supercritical drying has been published. “Ambient-pressure drying” techniques can be applied on a large scale for industrial purposes. Many studies have synthesized water glass based aerogels using an ambient pressure drying method [29,30,31,32,33,34,35,36]. However, the transparency of such aerogels is still lower than that of aerogels produced under high pressure [37]. Shrinkage during ambient pressure drying can be minimized by enhancing the stiffness of the gel network. The reinforcement the gel network can be achieved by several routes; one of them is the addition of sodium silicate or tetraethoxysilane (TEOS) before drying [38,39,40,41,42,43,44]. Some studies reported a successful fabrication of crack-free silica aerogel monoliths by aging a water glass based wet gel in a TEOS/EtOH solution prior to the solvent exchange and surface modification processes [33,34,35]. Another method is based on a passivation of the pore surface, inside the gel. Such a passivation can be induced by silylation for instance with trimethylchlorosilane [31] or hexamethyldisiloxane [14]. A preparation method rests on ion exchange treatments prior to drying [45,46].

Another way to avoid liquid-vapor interfaces is the application of “freeze-drying” methods. In this technique, the liquid must be first frozen and then sublimed. The pore liquid is frozen and then sublimed under vacuum. The “freeze-drying” method produces porous materials initially known as cryogels, now also termed aerogels. For application of freeze drying, the solvent of wet gel must be replaced by one with a low expansion coefficient and a high sublimation pressure. The main disadvantage of the freeze drying technique is that the gel network may eventually be destroyed by the nucleation and growth of solvent crystals, which can result in small dried particles and very large pores [45,47,48,49,50].

## 3. Influence of Synthesis Conditions on Nanoporous Structure of Silicate Aerogels

Numerous investigations have shown that variations in synthesis conditions (e.g., the type of precursors, catalyst, and surfactants; the ratio of water to precursor; the concentrations; the medium pH; and the solvent) modify the structure and properties of aerogels [51,52,53,54,55,56,57,58,59,60]. Synthesis parameters influence both hydrolysis and condensation rates and thereby regulate the kinetics and mechanism of sol–gel process. The most important factor for the final structures is the relative rate of the hydrolysis and condensation reactions. The relative rate of the simultaneous hydrolysis and condensation processes is dependent on the chemical and quantitative composition of initial solutions. The solution technique of sol-gel method gives much flexibility in adjusting the hydrolysis and condensation reaction rates, hence, to tailor the texture of gels. The addition of modifying agents can also have a strong effect on the final structure of aerogels. Special attention to silica based mixed oxide aerogels is given in this paper, *i.e.,* to the effect of introduction of a metal ion into the silica network.

### 3.1. Type of initial materials

Several excellent studies have been reported on the influence of the starting materials; illustrating some examples for that: Schubert, Hüsing in 2000 [25]; Pierre and Pajonk in 2002 [45]; Einarsrud *et al*. [61]; Rao *et al*. [62], *etc*.

Summarizing these studies, the most common silicon precursors are the alkoxides, Si(OR)_4_. The Si-O-R bonds are subjected to hydrolysis reactions during the gelation, which lead to a replacement of OR ligand by OH one in a nucleophilic substitution. Condensation reactions characterized with also nucleophilic nature follow the hydrolysis. Lower alkoxy groups such as methoxy, ethoxy, *n*-propoxy or isopropoxy are preferable as alkoxy group in the starting silane compounds [63]. Any branching of alkoxy groups or lengthening of the chain slows the hydrolysis rate of the alkoxysilanes. The reaction rate decreases in the next order: Si(OMe)_4_ > Si(OEt)_4_ > Si(O*^n^*Pr)_4_ > Si(O*^i^*Pr)_4_ [25]. The low hydrolysis rate limits the particle nucleations and the hydrolyzed species can be aggregated to larger particles resulting in lower surface areas and higher pores. As an example of the alkoxy effect; comparing the aerogels obtained from various Si alkoxides, tetramethoxysilane [Si(OMe)_4_] can yield more narrow and uniform pores and higher surface area than tetraethoxysilane [Si(OEt)_4_] [64].

The electron density at the silicon atoms, which has an important role in the nucleophilic attack, decreases in the following order: ≡Si−R > ≡Si−OR > ≡Si−OH > ≡Si−O−Si [25]. Thus, the Si−R bonds are very reactive, and show the least resistance to nucleophilic attack. The reaction rates of hydrolysis and condensation under acidic conditions increase in the same order as the electron density. By replacement of OR by R on silicon atoms, the relative rates of hydrolysis and condensation processes can be easily regulated. The enhancement in the number of alkyl groups reduces the bulk density and the volume of shrinkage of aerogels [63,65]. The use of methyltriethoxysilane (MTES) produces a more flexible network and higher surface area than TEOS [66]. Lower MTES contents give bigger and non-spherical particles, while higher MTES contents produces smaller and more spherical particles with a more uniform size distribution [66]. Highly flexible and superhydrophobic silica aerogels can be synthesized from the silicon precursor methyltrimethoxysilane (MTMS) [67]. A study of tetramethoxysilane (TMOS) and methyltrimethoxysilane (MTMS) mixtures has proved that the increase of the MTMS/TMOS molar ratio is correlated with an increase in surface area and a decrease in average pore size [68]. Addition of MTMS to TMOS or dimethyldiethoxysilane (DMDES) to TEOS increases the hydrophobicity of the aerogel and shifts the pore size distribution towards larger pore radii [69,70].

In recent years, many investigations related to the application of polyethoxydisiloxane (PEDS) have been published. The purposes of these studies are usually to reduce the cost of aerogel procedure [71] and to create hydrophobic aerogel surface [72]. The ultralow density aerogel prepared from PEDS has a chain-like microstructure and the average width of the chains is about several nm [73]. The elementary unit of the ultralow density silica aerogel is bigger (13 nm), the specific surface area is smaller (300–400 m^2^ g^-1^) than that of aerogels prepared with the conventional two-step processes. The pore size is about several nm [73]. The specific surface area, the pore volume and the average pore diameter are reduced via the density of silica aerogel. By applying ethanol as solvent, the lowest density can be achieved [73]. Zhou *et al*. synthesized hydrophobic aerogels from PEDS and perfluoroalkylsilane (PFAS) as a coprecursor [74]. The largest surface area (~1,100 m^2^ g^-1^) has been obtained at PFAS/PEDS volume ratios of 0.6.

The least expensive silicon precursors are aqueous solutions of sodium silicate (water glass), which are only stable under strongly alkaline conditions. Sodium silicate solutions are hydrolyzed in water or acidic solution and result in silicic acid colloid solutions, which can be gelled by lowering the pH. The gelled systems consist of different silicic acid particles (H_2_SiO_3_, H_2_Si_2_O_5_, H_4_Si_3_O_8_ ∙ xH_2_O *etc*). The Na_2_O/SiO_2_, *i.e.,* Na_2_SiO_3_/H_2_O mole ratio and silica content are important parameters for the sol-gel preparation from water glass solution. The optimal hydrophobicity and physical properties can be obtained with Na_2_SiO_3_/H_2_O molar ratios of > 8 × 10^−3^ [62]. According to Hwang *et al*. the best value of Na_2_O/SiO_2_ molar ratio is 1/3.3 and the best silica content is 4–8% in respect of the optimal aerogel properties [75]. Investigations of aerogel structures synthesized from different precursors have been reported by Einarsrud *et al*. [61]. The aerogels produced from water glass solution present the highest degree of monolithicity, the highest stiffness and the largest pore size [61]. In the case of the alkoxysilane precursors, both hydrolysis and condensation reactions run simultaneously. With sodium silicate, only condensation reactions should be taken into account [25,45].

### 3.2. Effect of pH

The influence of pH on the structure and properties of aerogels has been studied in great detail [24,25,45]. A brief description of some significant aspects will be presented here. The relative rate of hydrolysis and condensation reactions, which has the most important effect on the final structures, can be very effectively influenced by pH. The lowest reaction rate for hydrolysis is at pH = 7 and for condensation around 4.5. Under acid-catalyzed conditions (pH < 5), the hydrolysis is favored, the condensation reactions are limiting. The initial molecules form first many small oligomers and particles with reactive Si–OH groups, after that linear or randomly branched chains. The polymer-like networks contain small pores. The acid-catalyzed gelation is primarily characterized by a cluster-cluster growth model [24,25,76]. The kinetics of aggregation may be limited by the rate of condensation (e.g., reaction limited cluster aggregation model, RLCA [25,77]) or by the rate of diffusion (e.g., diffusion limited cluster aggregation model, DLCA [78]). Brinker and Scherer have described how the condensation takes place according to the cluster-cluster growth model under acidic (pH ~2.4) conditions, resulting in linear polymeric complexes with high surface area and low specific pore volume [24]. Above pH = 2.4, the rate of hydrolysis reaction reduces and the rate of condensation will be higher, the number of siloxane bonds (Si–O–Si) is growing. The growth mechanism changes from cluster-cluster to monomer-cluster [79]. As a result, more branched structures are formed, which can be characterized by increasing pore volume and lowering specific surface area.

Under basic conditions (pH > 7), the hydrolysis and particle nucleation processes are rate-determining and the condensation processes are dominant [24,25]. So the molecules of precursors are aggregated to fewer, larger, and denser particles than at low pH. The larger particles result in smaller surface area and larger pores [25,80]. Condensation of clusters with each other is relatively unfavorable [25]. More branched networks are obtained under basic conditions and rather chain-like network under acidic conditions. The reason of that is the condensation reaction on the network former silicon atoms⌠Si−(O−Si)_2–3_⌡, which are favored at high pH. The reactions on the terminal atoms ⌠Si−(O−Si)_1_⌡ have important role at low pH. The silica aerogels are usually prepared by base-catalyzed sol-gel method from silicon alkoxides, mostly with ammonia as catalyst. The surface area extends from 428 m^2^ g^-1^ at pH = 5.9 to 1082 m^2^ g^-1^ at pH = 7.3. Further increase of pH up to 8.6 results in a reversed effect; the surface area reduces by ~270 m^2^ g^-1^ [79]. Base-catalyzed condensation reaction leads to stiffening, which stabilizes the gels and produces an aerogel with a low density of 0.004 g cm^-3^. The typical values of aerogel density are 0.030–0.300 g cm^-3^ [81]. The reported growth models for base-catalyzed gelation are not unified. The monomer-cluster kinetic model seems to be mostly substantiated for base-catalyzed condensation [24,82]. Beyond the reaction limited monomer cluster growth theory (RLMC), the Eden growth model can be mentioned for description of gelation process [25].

### 3.3. Effect of catalyst

The effect of catalysis is strongly connected to that of pH, because the catalyst for sol-gel methods is typically an acid or a base. Acid catalysis can be performed using HCl, H_2_SO_4_, HNO_3_, HF, oxalic, formic or acetic acids. The base catalyst is usually NH_3_ or NaOH. It is well known that the catalysts greatly influence the physical properties of silica or silicate aerogels by means of theirs effects on pH [83,84,85,86,87]. In the case of acid catalysis, a typical volume ratio of TEOS/C_2_H_5_OH/H_2_O/acid is 1:10:4:0.01 and at basic catalysis a usual molar ratio of TMOS/MeOH/H_2_O/NH_4_OH is 1:12:4:0.005, respectively [88]. The type of acids does not have a dominant effect on the structure, but in a study of various acid catalysts it was observed that HF catalysis yields the highest pore volume and pore diameter but the gel proved to be weak [89]. Investigating the effect of acid concentration, only turbid colloid solutions can be obtained below 0.005 N HCl or HNO_3_ concentrations due to the insufficient amount of catalyst, *i.e.,* the incomplete hydrolysis of TEOS [90]. Clear and transparent alcogels are formed with the acid catalyst concentrations above 0.008 N. It was found that the higher the concentration of catalyst, the larger the silica aerogel density is [90]. In the case of NH_4_OH, Rao and Parvathy have specified that clear silica alcogels can only be obtained at 0.01 N concentration of catalyst [90]. At concentrations of NH_4_OH higher than 0.01 N, only turbid and opaque colloid solutions (sols) arise. This result may be explained by the reaction of TEOS with the basic reagent NH_4_OH.

Sol-gel catalysts without any acidic or basic features are usually nucleophiles. The nucleophilic catalyst generates pentacoordinated silicon intermediates, which are more reactive than the tetracoordinated silicon atoms. The pentacoordinated silicon precursor molecules can be more easily subjected to substitutions in hydrolysis or condensation reactions. *N*-methylimidazole, hexamethylphosphoric triamide, *N,N*-dimethylaminopyridine, SnBu_2_(OAc)_2_ or fluorides are some examples of these nucleophilic catalysts [91]. The presence of certain metal complexes can also effect the gelation processes, for example, the strong oxidant cerium(IV) and lanthanide complexes [91].

### 3.4. Effect of precursor concentration

According to the published results, the bulk density of aerogels is gradually enhanced with increasing precursor concentration in the initial and aging solution of sol-gel technique. Gels prepared with lower amounts of solvent have a higher density. The higher concentration of precursors supports the condensation reaction, because the larger amount of solvent separates the reacting species from each other. At lower precursor concentration, the hydrolysis reactions are favoured rather than the polymerization. Thus, the polymer size decreases. Comparing the influences of precursor, water, and polymer concentration, the density significantly will be higher with enhance of these concentrations, however, the precursor concentration has the most predominant effect on the density and the pore size [92]. For example, using TEOS as silicon precursor and ethanol as solvent, clear sols and gels are only formed above two molar ratios of EtOH/TEOS [90]. Higher molar ratios of EtOH/TEOS result in lower density, greater porosity of the aerogels and better transparency [90]. Another example, the specific surface area and pore volume of silica aerogel drastically diminish above precursor concentration of 15–25 wt % and the bulk density increases [92]. The compact aerogel structures can be characterized by particles of smaller sizes, low pore volume, relatively low specific surface area, and probably partly closed pores [79].

### 3.5. Effect of water content

The amount of water used in the initial gelation solutions can significantly affect the silica framework. At lower molar ratios, the H_2_O is not sufficient to complete the hydrolysis reaction of the silicon precursor [24]. The molar ratio of H_2_O/Si(OR)_4_ in the gelation solutions should be at least 2:1 to approach the minimal hydrolysis degree of the alkoxide required for the gelation. Ratios of water/alkoxy group ≤ 2 favor the condensation reactions. In the case of silicon alkoxide precursor, the incomplete hydrolysis leads to linear chain formation with residual organic groups [90]. On the other hand, at higher molar ratio, the hydrolysis proceeds faster, and the condensation takes place slowly. Water/alkoxy group ratios > 4 induce very loose gel networks with high porosity and smaller particles. Due to the presence of excess water, the rate of polymerization is lower than that of condensation, producing cyclization and enhancing the siloxane bond formation within the particles [90]. At molar ratios lower than 2 and higher than 12, only dense and cracked aerogels will be formed. The lowest density (0.08 g cm^-3^) and the most transparent (90%) aerogels can be obtained with molar ratios between 6 and 10 [90]. In another study, the highest surface area (~1,000 m^2^ g^-1^) could be achieved at stoichiometric amount of water (molar ratios ~4) [79]. Both deficiency (−20%) and excess (+20%) of water result in a reduction of surface area by 200–300 m^2^ g^-1^. In this range of water content, the bulk density of aerogels change slightly; only by > 30% excess of water it drops drown from 0.085 to 0.074-g cm^-3^.

The studies of mutual influence of water content and pH can be summarized as follows: at molar ratio of water/silicon precursor ~4 and pH < 2, the condensation of completely hydrolyzed species can be characterized by reaction limited cluster-cluster aggregation (RLCC), producing weakly branched structures [93]. Under acidic and low water conditions (molar ratio of water/silicon precursor < 4), the condensation of the incompletely hydrolyzed species is also expected to occur by RLCC aggregation. Due to the reducing effect of OR groups on the functionality of the condensing species, the structures will be more weakly branched [93]. At the pH ~7 and high water content, the growth of gel networks occurs primarily by reaction limited monomer-cluster aggregation (RLMC) and compact, non-fractal structures will be formed. At pH ~7 and low water content, the non-hydrolyzed species are incorporated into the growing clusters and the gelation can be described by a “poisoned” Eden model [93].

Investigating the effect of cross-linking agents together with the water content, the surface areas for monoliths with uncross-linked network reduce with decreasing water concentration, whereas the surface area for cross-linked monoliths show an opposite trend [92].

### 3.6. Effect of solvent

The polarity, the viscosity, and the protic or non-protic behavior of the solvent influence the reaction rate, and thereby the structure of the final materials. The typical polar and protic solvents applied in sol-gel methods are H_2_O, alcohols; a typical example of a polar but non-protic solvent is acetone; and tetrahydrofuran, dioxane or cyclohexane can be non-polar solvents. The polar and protic solvents stabilize the polar siliceous species such as [Si(OR)_x_(OH)_y_]_n_ by H-bridges. The non-polar solvent may be used for organoalkoxysilanes or incompletely hydrolyzed systems [24]. The structural modification by solvents with high molecular weight and longer chain lengths is responsible for the low density, high porosity and more pore volumes in the silica aerogels. By increasing the number of carbon atoms in alcohols (e.g., from ethanol to penthanol), the specific surface area of aerogels is gradually expanding (e.g., in the case of silica from ~300 m^2^ g^-1^ to ~450 m^2^ g^-1^); the porosity is increasing (from 40 to 45%); and the average pore diameter will be reduced (e.g., from 4.1 to 3.1) [68]. Supporting these results, the use of isopropanol produces less volume of shrinkage in the gels during the drying; and transparent, highly hydrophobic silica aerogels with low density (0.07 g cm^-3^), high porosity (>90%) and low thermal conductive (~0.09 W/mK) are formed [36].

Applying a mixture of solvents can finely regulate the structures in the gelation procedures. This review provides some examples for that. The aerogels prepared in pure water from silicon alkoxide precursors contain primary particles with smooth surfaces. In the presence of methanol-water mixtures, the primary particles will be monodisperse and form a fractally aggregated network [94]. The presence of methanol limits the growth of primary particles to submicron sizes. In another investigation it was found that the density and the volume of shrinkage can be reduced with ethanol and hexane mixtures [95]. The presence of low dielectric constant solvents (benzene, toluene, or anisole) in varying ratios with methanol (or other alcohols) leads to a rapid gelation and a formation of mesoporous materials with fibrous, open web-like structures, high surface area, and large pore volume [96]. The presence of high dielectric constant (polar non-protic) solvents such as acetone, acetonitrile, DMF, DMSO, or dimethyaniline have no such effect [97]. A partial charge model may be employed to explain the effect of low dielectric solvents on the rate of hydrolysis. By this model, the hydrolysis step will be rate determining, approaching diffusion control in this gelation process [96]. The dilution of methanol by less polar organofunctional alkoxysilanes (e.g., MTMO, MEMO) may decompose the hydrogen-bond network and changes the relative rates of hydrolysis and condensation reactions [99]. The hydrolysis/condensation ratio will be obviously higher by addition of organofunctional alkoxysilanes. The silanes will probably accelerate the hydrolysis reaction. Cluster-cluster aggregation provides the most acceptable growth model in this process [98].

The density of the final aerogel can be controlled by varying the ratio of solvent to precursor in the initial solutions. The amount of solvent defines the pore volume within the silica network. Thus, a high solvent to precursor ratio could result in a low density silica aerogel, similar to the effect of excess water content [77,97,98]. For example, the rise of solvent content by about sixfold leads to around threefold growth in the specific surface area at aluminosilicate fractal systems [98]. Using an alcoholic medium, a large amount of alcohol can slow the condensation processes by esterification (replacement of -OH with -OR) and can promote breaking the siloxane bonds by alcoholysis (≡Si−O−Si≡ + ROH → ≡Si−OH + ≡Si−OR). Alcoholysis and esterification induce to weaken the gels and some shrinkage [94]. The solvent content affects less the nanostructure; the degree of branching in the network and the size of primary particles [98].

Sonochemistry is an alternative method to promote the hydrolysis of alkoxy precursors without using alcoholic solvents [100,101,102]. The initial precursor-water solutions are subjected to intense ultrasonic irradiation. The absence of alcoholic solvents and the effect of ultrasonic waves provide the gel systems with unique features such as high density, fine and homogeneous texture, *etc*.

### 3.7. Effect of modifying agents

The structures, and thereby the properties of aerogels, can be modified by treatment of silica aerogels with different chemical agents, which are mainly organic entities. Controlled processes of inorganic precursors in the presence of functionalizing agents are required to achieve both a tailored microstructure and porosity. The modification of silica aerogels is generally carried out with various types of organic molecules; such as surfactants, cross-linking agents, or organic templates. The polymers applied in the preparation of organic-inorganic hybrid aerogels form a separated category. The treatment by chemical agents can be performed with various routes. Application of a postsynthesis treatment, e.g., doping or dipping of aerogels in organic solutions is limited. A more favoured route for the treatment is to use hydrolysable and condensable silicon precursors modified with organic groups. The organic group is covalently attached to the silicon atoms. In this route, the organic molecules can be already incorporated into the silica network during the sol-gel processing. The studies of the influence of chemical agents on the aerogel structures cover a very wide range of the materials, therefore only a couple of examples can be presented here.

The modifying agent applied usually is polyethylene glycol (PEG) [103,104,105]. High concentrations of PEG weaken the solid matrix, whereas low concentrations of PEG strengthen the matrix [105]. PEG acts as a through-pore template and a solubilizer of the silane reagent [104]. Narrow and more uniform pore size distributions can be obtained with addition of glycerol. Nowadays a lot of studies are dealing with silica aerogels cross-linked by isocyanate [106,107,108]. Di-isocyanates react with silanols on the surface of wet gels before supercritical drying and result in a significantly increase in the strength of aerogels and slightly affect the density or porosity [106]. The functionalized silanes such as aminopropyl-(triethoxy)silane [109]; 3-(2-aminoethylamino)-propyl-(trimethoxy)silane [110]; *N*-octyl-(triethoxy)silane [111]; 2-phosphinoethyl-(trimethoxy)silane or 3-carbamatopropyl-(trimethoxy)silane [99] provide examples for the organofunctional alkoxysilanes precursor [R`Si(OR)_3_]. Increasing the portion of R`Si(OR)_3_ in the starting mixture forces the shrinkage, but does not effect the density [99]. The organically modified samples also have open, cylindrical pores, however the specific surface areas are significantly lower than for an unmodified silica aerogel. The organic modification generally enlarges the particle sizes. The fractal dimension *D*, which indicates the degree of aerogel network branching, will be larger with the higher surface area; *i.e.,* with the reduced portion of R`Si(OR)_3_ [112]. The influence of the functional organic group on the fractal dimension may be caused by strengthening or weakening the hydrogen-bond network [113]. The effect of organofunctional groups (R`Si) can be explained with their more reactive character. The type of organofunctional groups has generally no effect on the structures [99].

**Figure 2:**
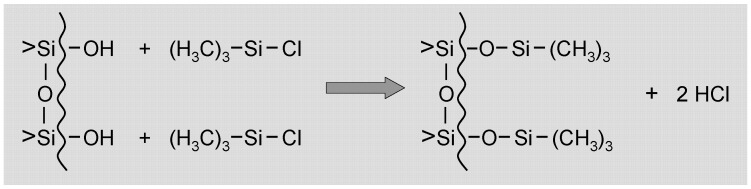
Reaction of surface silanol groups with TMCS.

*Surfactants* constitute a separated section of modifying agents. Surfactants are typically applied in sol-gel techniques to minimize the shrinkage, prevent cracking and avoid supercritical drying processes. The surfactants decrease the capillary stress. The alco- or hydrogels can be made hydrophobic by silylation, which changes the liquid-solid contact angle and annihilates the capillary liquid tension. Various kind of materials are used as surfactant in the sol-gel processing: trimethylchlorosilane (TMCS) [75,114,115,116,117,118,119]; hexamethyldisiloxane (HMDS) [75,115,116,117,118,119]; hexamethyldisilazane (HMDZ) [115,119]; triblock co-polymers (Pluronic-P123; ethylene oxide, propylene oxide) [120]; hydroxypropyl cellulose (HPC) [121]; cetyltrimethylammonium bromide (CTAB) [122]; polyoxyethylene sorbitan trioleate (Tween 85), a nonionic surfactant [123], and so on. One of the most favourite sol-gel surfactant is trimethylchlorosilane (TMCS) [114]. Using TMCS, the elimination of capillary stress can be performed by pore water solvent exchange and surface modification of wet gel before ambient drying. The TMCS influences by its interaction with pore water and Si–OH groups on the surface of wet gels. In this process, the chloride groups will be substituted by OH groups. The reaction between TMCS and pore water is very rapid, in order to slow this process, ethanol or another alcoholic solvent is used, which reacts also with TMCS in a substitution step. The process between TMCS and alcohol decreases the reaction rate of TMCS with pore water, which is favorable for achieving crack-free aerogels. The active OH groups will be converted into inactive O−Si−R ⌠e.g., O−Si−(CH_3_)_3_⌡ groups (Figure 2), resulted in gels with zero capillary force during drying [118].

Low density aerogels (0.1–0.3 g cm^-3^ [115]; ~ 0.04 g cm^-3^ [119]) can be prepared by using TMOS and/or water glass in the presence of TMCS and HMDZ silylating agents [115] or TMCS + HMDS [119] as surface modifiers. In these systems, HMDZ results in the best quality of silica aerogels in respect of monolithicity and visual transparency, however, the bulk density is high (~0.1 g cm^-3^) and the dried gels result in many cracks. The lowest density (~0.04 g cm^-3^), the highest optical transmission, and low thermal conductive (0.047 W/m K) can be achieved by using TMCS + HMDS mixture [119]. By HMDS surface modification, hydrophilic and high density aerogels are formed. The porosity is about 98% in the case of TMCS and 95% at HMDZ. The transparent is less (~10%) at TMCS and higher (~70%) at HMDZ, respectively. The use of HMDS and HMDZ leads a non-complete surface coverage [115].

Comparing studies of silicate aerogel structures obtained with and without surfactant prove that the application of surfactants and surface silylation agents (TMCS, HMDS, Brij 56, P 123) condense the fractal or branched structures and the elementary units, thus, significantly reduces the porosity. For example, the use of surfactants or surface silylation agents (TMCS, Brij 56, P 123) reduces the specific surface areas from ~650 to 110–150 m^2^ g^-1^ in the case of aluminosilicate systems starting from Al nitrate and TEOS [98]. The surfactants cover and smooth the surface of particles and displace other molecules, ions, which can be attracted to the surface of particles and contribute to formation of loose structures.

### 3.8. Addition of polymer

Hybrid materials provide an attractive kind of organically-modified, gel-derived systems. In these materials, organic polymers are chemically incorporated into the inorganic network at a molecular level. The hybrid aerogels will be briefly mentioned here, because the topic is very broad and a book titled “*Inorganic-Organic Hybrid Porous Materials*” has been published recently [124]. One of the very important advantages of the sol-gel method is its capability to prepare numerous types of new organic-inorganic hybrid materials, which are either impossible or are extremely difficult to synthesize by any other processes. The fragility of aerogels hinders their exploitation. Thus, one of the most significant aims of aerogel researches is to greatly reduce the brittleness and rigidity of inorganic aerogels. Creating hybrid aerogels (e.g., Ormosils) offers a good way in realizing this aim. The aim of modifying silicate aerogels with organic groups or molecules is to supplement new properties without influencing the existing positive properties, such as good thermal insulation, transparency, and high surface area. The hybrid materials have unique mechanical properties, e.g., low elastic modulus and high ductility combined with high mechanical strength. The reported preparative methods describe various synthetic routes for linking inorganic and organic components. The organic initial materials may be used as monomers [125,126], oligomers [127,128,129,130], or cross-linked polymer networks [131]. Monomers (e.g., alkoxides) [126], nanoparticles [132,133,134], or macroscopic, preformed porous networks (e.g., aerogel particles) [134] can provide the inorganic components in the procedure. The most usually polymer in hybrid materials is polydimethylsiloxane (PDMS) [127,128,135,136,137,138,139]. PDMS improves the mechanical behaviour, the flexibility, formability, diminishes the brittleness [135]. As to porous structure, the application of PDMS strongly reduces the specific surface area and results in high pore size uniformity and pore interconnectivity [136]. The degree of decrease depends on the PDMS content; the larger the PDMS amount, the lower the specific surface area is [98,136].

The *template-based* organic-inorganic porous materials are worth emphasizing. The template-based organic-inorganic porous materials belong to the category of future developments, such as the synthesis of hierarchical morphologies that mimic the complicated structures found in Nature. Organic templates are introduced into a sol-gel matrix in order to tailor the pore size and volume. The removal of these templates creates pores that appear to reproduce the size and volume fraction of the added template phase. In contrast to conventional silica aerogels with less controllable and polydisperse porosity, e.g., a tunable, unimodal (nano)porosity can be achieved by templates. The pore volume and the pore connectivity depend on rather the volume fraction of template phase. The materials with controlled porosity are capable of selective adsorption or catalysis.

The tailored porosity can be formed by means of molecular-engineered materials. One of the most efficient and well known molecular-engineered materials are the bridged polysilsesquioxanes. Bridged polysilsesquioxanes are a family of hybrid organic-inorganic materials. The initial material of sol-gel technique contains a variable organic bridging group and two or more trifunctional silyl groups; (RO)_3_Si−R**′**−Si(OR)_3_, where *R***′** may be alkylene, arylene, alkenylene, or organo functionalized groups [124,140,141,142,143,144,145,146,147]. The various organic bridging groups are incorporated into the network. One of the first examples for organic bridging groups is telichelic polyisoxazoline, terminated with triethoxysilyl groups, which can be co-condensed with TEOS [140]. The gelation generally occurs quickly and at lower concentrations than for conventional silica gels. Furthermore, polysilsesquioxane gels can be synthesized with compositions that are inaccessible by sol-gel polymerization of organotrialkoxysilanes. Sol-gel polymerization of poly(trialkoxysilyl) monomers inherently leads to development of a 3-D network, *i.e.,* to formation of bridged polysilsesquioxanes. The organic component from the hybrid silicate-polyisoxazoline gels can be oxidatively removed, leaving a porous silica matrix. The type of *R***′** groups effectively influences the nanostructure of hybrid aerogels. The specific surface area for arylene-bridged polysilsesquioxanes varies between 750 and 1,200 m^2^ g^-1^ and the mean pore diameter between 6 and 34 nm. The alkynene-bridged polysilsesquioxanes can be characterized with 450–650 m^2^ g^-1^ surface areas and 20–50 nm mean pore diameters depending on the length of alkylene groups. The specific surface area is decreased with lengthening alkylene chains. The bridging group also can control the size of pores. More recently, studies have focused rather on building functionality into the bridging groups [142]. Shea and Loy varied the organic groups which are covalently attached to the trifunctional silicon groups through Si−C bonds. The length, the rigidity, the geometry of substitution, and the functionality of organic chains can be modified. This variability provides an opportunity to tailor the properties such as porosity, thermal stability, refractive index, optical clarity, chemical resistance, hydrophobicity, and dielectric constant [142]. Precise levels of control have been also achieved. Nowadays the studies are ongoing with e.g., an inorganic silsesquicarbodiimide network of the type [(NCN)_1.5_Si-(CH_2_)*_x_*-Si(NCN)_1.5_]*_n_* [144] or coumarin-dimer-bridged polysilsesquioxanes [147]. Microporous organic-integrated silica synthesized from alkylamine as surfactant and bis(triethoxysilyl)ethane as network former reveals periodic and uniform pore sizes of 1–2 nm [148]. Using another surfactant, a large block copolymer together with bis(triethoxysilyl)ethane produces a well-ordered mesostructured silica aerogel with 10 nm pores [149].

There are examples for other type template-based organic-inorganic porous materials. The surfactant-templated silica aerogel prepared with CTAB is characterized by low bulk density (<0.1 g cm^-3^); less specific surface area (~700 m^2^ g^-1^); high volume fraction porosities (>95%) of the hexagonally packed pores; and negligible shrinkage [94]. 4,4’-bis(Triethoxysilyl)biphenyl [150]; polyimides [151]; and a novel room-temperature ionic liquid (RTIL), [C_4_mim]^+^ BF_4_^-^ [152] have been applied as templates to produce monolithic mesoporous organic-silica hybrid materials.

### 3.9. Incorporation of metal ions

Only limited research data are available for the influences of various metal ions which are incorporated into the silicate network and much less concerning the comparison of the structures of mixed oxide aerogels to those of pure silica aerogels.

The metal (M) precursors may be alkoxides [M(OR)_z_] or inorganic salts [M(NO_3_)_z_, MCl_z_]. The general problem in applying mixtures of two or more alkoxide precursors is the different hydrolysis and condensation rates. Regarding the nucleophilic character of hydrolysis, the mechanisms depend on the partial positive electronic charge, *δ*^+^ on the metal atoms. Many metal atoms such Al, Zr, and Ti are much more reactive towards water than alkoxysilanes due to their significant positive partial charges. The positive partial charges are derived from lower electronegativity and higher Lewis acidity of metal atoms. Both the hydrolysis and condensation reactions of Al, Zr or Ti precursors are fast. The silicon atoms carry substantially less positive charge, thus the hydrolysis and condensation reactions of silicon alkoxides occur at much lower rates. The network former character of Si or other metal atoms depends on the ionic nature of the Si-O bond and the M-O bonds. The ionic character is increasing in the next order: SiO_2_ < Al_2_O_3_ < TiO_2_ < ZrO_2_ < Na_2_O [45]. The bonds of SiO_2_ can be characterized by about 50% covalent feature [45]. This order is correspond to the reactivity sequence of tetravalent alkoxides in hydrolysis reactions: Si(O*^i^*Pr)_4_ << Ti(O*^i^*Pr)_4_ < Zr(O*^i^*Pr)_4_ < Ce(O*^i^*Pr)_4_.

If precursors with dissimilar hydrolysis and condensation rates are reacted with each other, heterogeneous microstructures will be formed. The faster reacting component usually produces a separated phase such as sol particles. In order to avoid the formation of heterogeneous structures, the slower reacting precursor should be prehydrolyzed. Another possibility may be to reduce the reaction rates of the electropositive metal alkoxide precursors. The precursors can be modified, e.g., chelated with slowly hydrolyzing multidentate ligands such as acetylacetonate [153] or alcohol amines [154] [121,153]. The most frequently applied method is the addition of acetic acid or acetylacetone to the precursor solution, which results in partial substitution of the alkoxy groups. The chelate complexes have a different reactivity depending on the type and number of bidentate ligands. The complexation makes possible the chemical design of the precursors, so that the structure of the gel derived materials can be deliberately influenced.

**Figure 3:**
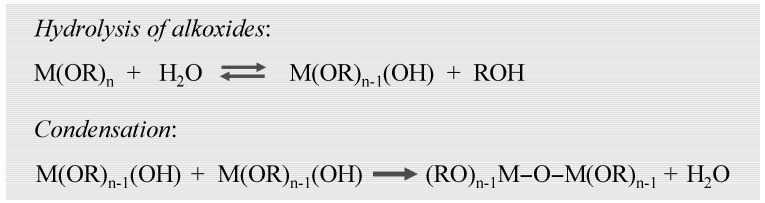
Gelation processes of metal alkoxides.

**Figure 4:**
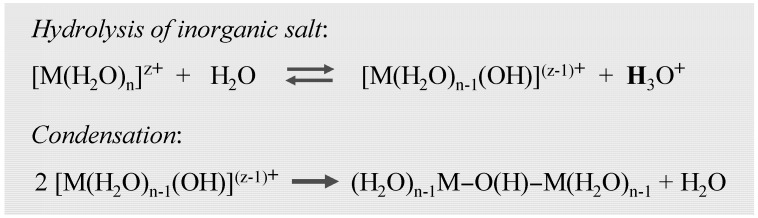
Gelation processes of inorganic metal salt.

In the case of alkoxy precursors, acidic or basic catalysts are required for the hydrolysis and condensation at the silicon alkoxides (Figure 3). Applying inorganic salts, the hydrolysis of metal ions produces and acidic medium, thus catalysis may be not needed (Figure 4).

#### 3.9.1. Incorporation of alkaline and alkaline earth metal ions

Silicates with basic character can be synthesized by incorporating basic species into silicate network. The basic species may be alkaline or alkaline earth metal oxides. Alkaline and alkaline earth metal ions may have only a network modifying role, as the ions cannot replace the silicon atoms in the Si–O–Si bonds. Thus, their incorporation is not a simple task. Since the alkaline earth alkoxides aggressively react with water and produce precipitates, the alkaline earth alkoxides must be directly synthesized in alcoholic gelation solutions from alkaline earth metals. The metals react with the alcohol in the solution of partially hydrolyzed species of R_n_Si(OC_2_H_5_)_4-n_ to give a stable mixture of alkaline-earth alkoxide and R_n_Si(OC_2_H_5_)_4-n_. HSi(OC_2_H_5_)_3_ applied in the sol-gel processing has a gelation accelerating role [155,156]. Several sol-gel methods have been published, which produce metastable homogeneous glasses being within the immiscibility region; e.g., SrO-SiO_2_ [159] and CaO–SiO_2_ systems [160,161].

*Lithium silicate* porous materials can be prepared by reaction of HSi(OC_2_H_5_)_3_ with aqueous lithium silicate solutions (SiO_2_/Li_2_O = 7.5) [156,162]. Not only ethoxy group, but also Si-H bonds are also hydrolyzed under the basic conditions of aqueous lithium silicate solutions. The gelation occurs immediately and the escape of hydrogen gas produces macroporous lithium silicate systems. The lithium silicate foams have a specific surface area of about 300 m^2^ g^-1^ and the average pore size is 20–30 nm (Table 1).

In the synthesisof *magnesium silicate* (forsterite), magnesium inorganic salt [Mg(NO_3_)_2_ [163,164,165,166] or alkoxide [Mg(OCH_2_CH_3_)_2_ [164,167] precursors may be used. The aerogels with 5 and 10% MgO provided by Mg nitrate present surface areas of 240 and 200 m^2^ g^-1^, respectively [165,166]. The adsorption measurements prove that the higher the MgO content, the larger the specific surface area is (Table 1). In the application of Mg(OEt)_2_, the TEOS precursor must be prehydrolyzed due to the different hydrolysis rate of TEOS and Mg(OEt)_2_. The forsterite gels dried supercritically are characterized by surface areas of ~520 m^2^ g^-1^ [167,168]. In the case of 2 MgO·SiO_2_ prepared from alkoxide precursors using various prehydrolysis conditions for TEOS, the aerogels have surface areas between 207 and 340 m^2^ g^-1^, 0.135 g cm^-3^ density, and loosely packed agglomerates with primary particle sizes between 10 and 15 nm [169]. A forsterite aerogel without supercritical drying can be prepared by using alkoxides modified with acetic anhydride and TEOS [170].

*Calcium silicate* aerogels have received more interest than the other alkaline earth metal oxide containing aerogels due to their bioactivity. In the presence of Ca and Ba metals, the gelation of hydrolyzed alkoxysilane [R_n_Si(OC_2_H_5_)_4-n_, HSi(OC_2_H_5_)_3_] leads to the formation of calcium and barium silicates, respectively [155,156]. The weak basic nature of calcium and barium silicates is due to the incorporation of basic Ca and Ba components into the silicate networks [156,157,158]. There are examples of the use of calcium nitrate [171] or alkoxide [172,173] precursors. Starting from calcium nitrate precursor, the first step is the synthesis of larnite (Ca_2_SiO_4_) powders by reactions of colloidal silica and calcium nitrate in the presence of ethylene glycol. Larnite powders modified with 3-aminopropyltriethoxysilane will add to a silica sol prepared from TEOS [171]. The specific surface area of larnite–silica aerogels gradually reduces with increasing the CaO content and the concentration of the active phase. For example, 44% of CaO content diminishes the 1,000 m^2^ g^-1^ specific surface area of pure silica aerogel to ~50 m^2^ g^-1^ [171]. The reducing effect of Ca ion can be attributed to its network modifying character, the Ca ions break the 3-D silica network. Xonotlite-type calcium silicate (6CaO ·6SiO_2_·H_2_O) synthesized by sol-gel technique from alkoxide precursors is a porous insulation material [172]. Xonotlite-type calcium silicate is made up of hollow spherical agglomerates and has excellent insulating properties, such as low thermal conductivity, environment friendly, high strength, and wide applying temperature range [172]. The density of xonotlite-aerogel is 0.1–0.13 g cm^-3^, the specific surface area is 200–300 m^2^ g^-1^. The mean pore diameter of aerogel is in the range of 10 to 50 nm [173].

Calcium-containing aerogels with desirable properties, e.g., excellent biological activity, can be obtained by preparation of CaO−SiO_2_−PDMS hybrid systems [125]. TEOS, silanol-terminated PDMS, and Ca(NO_3_)_2_ ·4H_2_O starting materials produce the biological active “sono-aerogels”. The high pore size uniformity and pore interconnectivity are characteristic for porosity. The diameter of pores is in the range of 2–200 nm; the density is close to 0.5 g cm^-3^. When the calcium content is increasing, the specific surface area and the average pore diameter are decreasing in this system. For example, 20% of CaO content reduces the 1,280 m^2^ g^-1^ specific surface area of pure silica aerogel to ~600 m^2^ g^-1^ [125]. At a constant calcium content, the specific surface area and the average pore diameter diminish with higher PDMS content; the change of PDMS amount from 10 to 50% leads to reduction in specific surface area from 1,100 to ~510 m^2^ g^-1^. The glassy matrix contains some poorly crystallized domains of CaSiO_3_ nanocrystals; the amorphous areas consist of silica network. Thus, the incorporation of Ca ions gives rise to the formation of crystallization nucleis.

#### 3.9.2. Incorporation of aluminum ions

**Figure 5:**
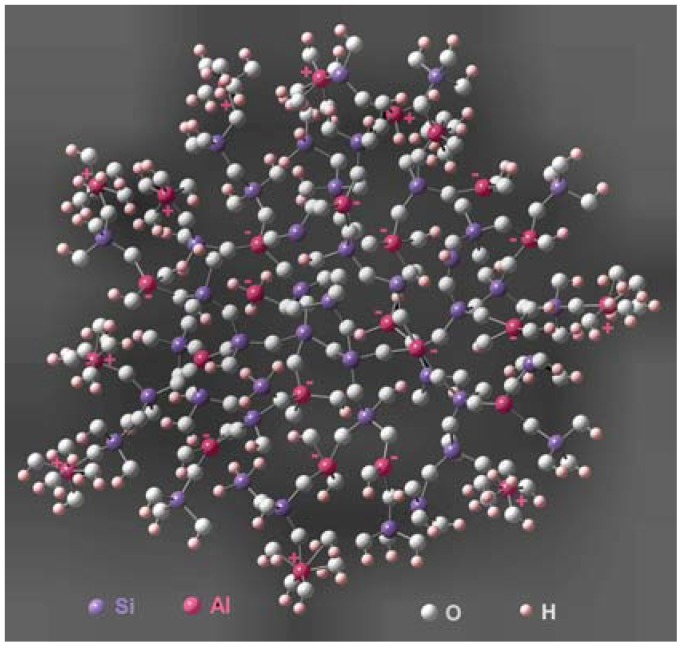
Chemical bond system of aluminosilicate aerogel with fractal structure. All geometry optimizations were performed using Gauss View. The size of elementary unit controlled by Gauss View is 3 nm [187].

Aluminosilicate aerogels are one of the most widely studied silica-based, mixed oxide aerogels. Sol–gel processing allows one to produce gel glasses with compositions lying within the liquid–liquid immiscibility dome of the Al_2_O_3_–SiO_2_ system. The aluminum ions can incorporate into the silica network in *octa*hedral as well as in tetrahedral sites. The function of octahedron Al ions is a network modifier and the positive charge of the octahedral Al(III) ions can compensate the negative charge of Al ions inserted in the tetrahedral sites as network formers (AlO_4_^−^) (Figure 5). The presence of octahedral Al ions allows higher Al incorporations than that obtained by traditional melting processes (10%). The octahedrally coordinated Al atoms are incorporated rather on the surface of elementary units, while the tetrahedrally coordinated atoms are mainly inside the elementary units. The octahedrally incorporated Al atoms are coordinated by water molecules and even by organic compounds in low fraction supporting the development of small elementary units and fractal structures., The silicon and aluminum oxides are always amorphous in mixed-oxide aerogels.

Several starting materials such as tetraalkoxysilane, water glass, organic aluminum salts (Al alkoxide [174,175,176,177,178,179,180,181,182], acetate [98]), and inorganic aluminum salts (Al nitrate [98,165,166,183,184], chloride [176,177]) can be applied in the wet chemical sol-gel method for preparation of aluminosilicate aerogels. The very fast hydrolysis of Al alkoxide precursors can be slowed considerably by complexation with a chelating agent such as ethyl acetoacetate [186] or another agent [179,180,181]. Several reported research data provides some evidences for the reducing effect of Al incorporation on the porosity of aluminosilicate aerogels prepared from alkoxy precursors (Table 1) [174,175,176,188]. According to another part of published results, the Al incorporation into the silicate network does not decrease the porosity in every case, and the influence depends rather on the type of precursors [98]. The starting materials significantly influence the gel structures [98,184]. The gel processing starting from Al nitrate or isopropoxide and TEOS yields aerogels with highest porosity. The large number of incorporated Al atoms into the silica network loosens significantly the fractal system by the molecules attracted on the surface of particles growing the specific surface area [98]. The fractal structure and the small elementary units guarantee the high porosity. The primary building units of the Al isopropoxide gels are much smaller and more compact than those of Al nitrate gel. The extensive hydrolysis of Al nitrate produces an acidic medium required for the hydrolysis of TEOS. The hydrolysis rate of Al nitrate is very similar to that of TEOS, which supports the Al incorporation into the silica framework. Using Al isopropoxide precursor, the TEOS should be prehydrolyzed if a homogeneous distribution is desired. The prehydrolysis time of TEOS determines the degree of the Al incorporation. With longer prehydrolysis time, the Al atoms are connected rather to the surface of elementary units, than to inside. The gel systems obtained from Al acetate and TEOS or water glass solutions present compact structures with small porosity (Table 1). The application of Al acetate requires a strong acidic medium, which drastically hinders the incorporation of Al(III) ions.

The aluminum content in the aluminosilicate systems is usually varied between 5 and 30% [174,177,188]. However, a number of examples for higher Al amount can also be found [189]. The aerogel with mullite compositions (e.g., 2 SiO_2_ ·3 Al_2_O_3_) is not typical. An example for the effect of aluminum content can be found in the study of aerogel synthesized from boehmite sol and TEOS. The density of silica aerogel changes from 0.040 to 0.52 g cm^-3^; the specific surface area from ~740 to ~420 m^2^ g^-1^ by incorporation of 15 wt% alumina (Table 1) [174]. The loss in the specific surface area and the total pore volume can be observed only at higher than 10 wt % alumina content.

Pierre *et al*. investigated the influences of chelation of the Al precursor and the rate of Al and Si precursors on the structure and found those have minor effects [173]. The complex formation with ethyl acetoacetate slows considerably the hydrolysis rate of aluminum tri-*sec*-butoxide precursor to that of Si alkoxide. The total pore volume of mesopores changes from 45% to ~75% at the Si/Al rate of 100 and pH ~2, while the effect is contrasted at pH ~0 [173]. The Si/Al rate roughly corresponds to the typical compositions of zeolites.

Tamon *et al*. compared the effect of aluminum and titanium ions [188]. According to their research data, the higher the alumina or titania, the lower the specific surface area is. The adsorption capacity of silica-alumina aerogel is larger than silica-titania aerogels; the changes in the specific surface area are very similar in both cases [188].

#### 3.9.3. Incorporation of transition metal ions

Oxide glasses containing transition metal oxide have been the subject of increasing interest, mainly owing to their semiconducting and catalytic properties. These properties can be attributed to the electron hopping between two different valence states of the transition metal ions. The metal oxides deposited on silica are generally considered as acidic types. Therefore, mixed oxides have been of great interest for use as catalysts owing to the variability of their surface acidity. In order to obtain desirable properties of these materials, a uniform and homogeneous distribution of the transition metal ions must be achieved in the silica matrix. The incorporation of transition metal ions into the silica network may be carried out by various routes. The use of sol–gel chemistry allows a large degree of influence over the extent of mixing metal oxide and silica. Several research data for sol-gel chemistry demonstrate developments of metastable homogeneous glasses being within the immiscibility region; e.g., SiO_2_–TiO_2_ [191]; yttrium silicate [192]. One route of sol-gel technique is the copolycondensation of silicon and metal alkoxide precursors. In this processing, the difficulty is to equate the hydrolysis rates of different precursors. Another preparation method may be the impregnation of silica wet or aerogels in a metal salt solution. In this route, the organometallic or coordination compounds of metals are attached to the pore walls of mesoporous silica gels by covalent bonds. The surface hydroxyl groups can react with the metal compounds. It is possibly to use inorganic metallic salt with crystal water content [e.g., M(NO_3_)_z_·m H_2_O] in organic solvents. The crystal water is required for the hydrolysis of the metal ions. The hydrolysis produces hydrogen ions and [M(OH)*_x_*(H_2_O)_n-*x*_]^(*z*-*x*)+^ ions, which complex metal ions are already capable for condensation reactions. An organic proton acceptor such as ethylene oxide, propylene oxide or an epoxide can be used to speed the hydrolysis [190].

*Silica-titania* mixed oxide aerogels are highly active catalysts owing to the pronounced mesoporosity and the high dispersion of titania in the silica network. The efficiency of silica–titania catalysts are strongly dependent on the molecular scale dispersion of titanium atoms, the high surface area, and pore diameters in the mesoporous range. In order to avoid the leaching of Ti ions from the silica skeleton, the hydrophilicity of aerogels must be modified. The active titanium species stay on the hydrophobic surfaces. A heat treatment at high temperature induces considerable segregations of titania and silica, which destroys the porous structure developed during the sol–gel process.

The literature reports several strategies for sol-gel preparing silica-titania aerogels. In one of the sol-gel routes, the silica alcogels are impregnated with titanium precursor producing aerogels with good mechanical strength [193,194,195,196]. The impregnated aerogel blocks exhibit less defects and the anatase TiO_2_ are deposited on the surface of silica networks. The slow diffusion of the titanium precursor into the alcogels requires a long impregnation time and leads to a nonuniform deposition of titania. The cohydrolysis of titanium and silicon precursors is another possible route to obtain crack-free, titania-silica aerogels [197,198,199,200]. The cohydrolysis process is more successful under basic conditions [197]. The generally used sol-gel Ti precursors are the alkoxides [202,203,205,206]. Prehydrolysis, complexation and/or polymer addition can be applied to adjust the hydrolysis and condensation rates of the silicon and titanium alkoxide precursors. The silica-titania aerogels synthesized by cogelation of alkoxides possess surface areas of 400–700 m^2^ g^-1^; pore volumes of 2–3 cm^3^ g^-1^; pore sizes of 10–30 nm; and densities in the range of 0.34–0.38 g cm^-3^ (Table 1) [202,203]. Low temperature supercritical drying has been reported to provide aerogels with lower microporosity, higher surface areas (up to 700 m^2^ g^-1^) and amorphous mixed oxide aerogels [201,202]. Deng [203] and Xu *et al*. [204] synthesized silica-titania aerogels with TiO_2_ /SiO_2_ molar ratio of 1:5, which possess high bulk mechanical strength and high porosity. The aerogels obtained from TEOS and titanyl sulfate precursors can be characterized by average particle size of ~10 mm and specific surface area of ~330 m^2^ g^-1^ [203,204].

Ti addition significantly modifies the pore structure of the gel systems. There is evidence that the incorporation of TiO_2_ into the gel network increases the specific surface area and the average pore diameter of aerogels [206,207]. Another investigation shows that the specific surface areas expands first by increasing SiO_2_ content and reaches a maximum (~ 620 m^2^ g^-1^) near the TiO_2_ / SiO_2_ molar ratio of 1/5 [203]. The pure titania aerogels only have a surface area of ~100 m^2^ g^-1^. The reduction of specific surface area by increasing titania content may be attributed to the weaker interactions between TiO_2_ and SiO_2_ in the titania–silica aerogels and to the higher coalescence of TiO_2_ gel particles [203]. Other researchers have found that the higher content of TiO_2_ yields smaller surface area in the silica-titania aerogels with any compositions [202,208]. The reduction in the surface area resulted by titanium incorporation can be attributed either to reduction in pore accessibility or to the occupation of titania in the pores of aerogels. According to Evans the Ti^4+^ ions are tetrahedrally coordinated in the silica matrix rather than octahedrally. Ti atoms are kinetically stabilized in tetrahedral coordination [209]. The XRD measurements verify the amorphous character of silica-titania systems up to 15 mol % TiO_2_ and 700 °C [210].

*Vanadia-silica* aerogels present semiconducting and photochemical properties. The presence of V_2_O_5_ inside the silica matrix results in electrical conductivity. Vanadium can be introduced into the silica matrix as a network former; however, a traditional melting process can yield only heterogeneous materials presenting a phase separation [211]. Several synthesis routes are reported for vanadia-silica aerogels. The impregnation with vanadium precursor can produce poor structures due to the weak interaction between vanadia species and silica-carriers [212]. Vanadia-silica mixed oxide aerogels have been prepared by sol–gel methods from various organic precursors, including vanadium(III) acetylacetonate, vanadium (V) oxide triisopropoxide or vanadium (III) acetylacetonate [212,213,214,215]. Vanadium(III) acetylacetonate, V(acac)_3_ is much less reactive with water than vanadium(V) oxide triisopropoxide, thus it is easier to handle and less toxic. In vanadylacetylacetonate, the oxidation number of vanadium is only +4 instead of +5 [212]. The sol-gel method renders possible to achieve a uniform and homogeneous distribution of vanadium ions in the silica matrix. Although, the vanadia-silica system has been reported as a mixed oxide rather than a vanadium silicate system [212]. Vanadia-silica aerogels derived from vanadium(III) acetylacetonate have similar textural properties to those of silica and exhibit a low vanadia surface concentration [215]. Higher vanadium surface concentrations can be detected in the samples derived from vanadium(V) oxide triisopropoxide [216]. Thus, the vanadia surface concentration can be efficiently tailored by type of vanadium precursors. The vanadium precursors influence even the porosity of the aerogels; the vanadium(III) acetylacetonate yields higher surface areas and pore volumes than vanadium (V) oxide triisopropoxide (~1,100 in contrast to ~ 650 m^2^ g^−1^; 3.4 in contrast to 1.6 cm^3^ g^−1^) [216]. According to other reported results, the cogelation of vanadylacetylacetonate and tetramethoxysilane produces lower porosity (BET surface areas of 530–540 m^2^ g^-1^ and mean pore sizes of 6.7–6.8 nm) [213]. There are examples even for the use of NaVO_3_ as vanadium precursor [217,218]. Vanadium pentoxide gel has been prepared from NaVO_3_ precursor, which can be combined with TEOS or methyltriethoxysilane in order to get highly branched vanadia–silica composite structures. The V_2_O_5_ polymeric chain may coexist with silica polymer and it can be homogeneously dispersed [217,218].

The effect of the V/Si ratio on nanostructures has been studied by several researchers [211,212,213,216]. The V_2_O_5_ content is usually varied in the range of 5–30 wt or 5–25 mol %. The structure and catalytic properties of the vanadia-silica mixed oxides were reported to be mainly influenced by the V/Si molar ratio and drying mode. The temperature of glass transition, *T_g_* is rising with the V/Si mole ratio [219]. The higher VO*_x_* content generally results in a decrease in the BET surface areas and the micropore areas [211,219]. For example, the BET surface area of the porous materials prepared from vanadium(III) acetylacetonate diminishes from 840 to 430 m^2^ g^-1^ as vanadia content grows from 10 to 30 wt % [212]. However, the reduction of surface area in the function of V/Si mole ratio is not consistent in every published investigation (Table 1) [219]. The reducing effect can be explained with blocking of the small pores. The layers of VO*_x_* may cover the pores and/or changes can occur in the speciation of the vanadium species [220]. Crystalline V_2_O_5_ can be usually observed in the aerogels which are dried under supercritical conditions [211,213]. Both the high-temperature supercritical drying and the high vanadia concentration induce the crystallization of V_2_O_5_. Several investigations could essentially identify amorphous phase even after calcinations at 500 ºC [219,221,222]. Drying procedure is a crucial parameter for controlling segregation-agglomeration and crystallisation of vanadia [216].

*Zirconia-silica* mixed oxide aerogel displays high thermal and chemical stability, improved mechanical strength, and active catalytic property owing to its strong surface acidity. Mixed oxides often show greatly enhanced catalytic activity compared with that of the individual component oxides. The porous materials with acid surface are one of the most important types of catalyst. The surface acidity derives from a charge imbalance resulted by the presence of minor component oxide, which rearranges the bond matrix of the major component [223]. The presence of ZrO_2_ increases both Lewis and Brønsted acid centers over ZrO_2_–SiO_2_ mixed oxide. The zirconium is responsible for Lewis acidity in ZrO_2_– SiO_2_ mixed oxide due to the higher ionicity of the Zr–O bond.

Applying the sol-gel technique, the hydrolysis level and acid or base catalysis can strongly influence the surface hydroxylation. The homogeneity of mixed oxides can be controlled by alkoxide ligand type, temperature of the reaction process, and precursor concentration [223]. Zirconium alkoxides [223,224,225,226,227] as well as zirconium nitrate [228] provide the Zr precursors in the sol-gel processing. Zirconium alkoxides are more reactive with water than silicon alkoxides, hereby the sol-gel derived aerogels with high Zr/Si ratios contain sometimes separated crystalline tetragonal zirconia phase. Prehydrolysis of silica precursor [223] or modification of Zr precursor (e.g., with methoxyethanol [226]) may be effective techniques to avoid the phase separation and the formation of mixed oxides with silica-enriched surfaces. Not only sol-gel processing, but the impregnation technique is also usually applied for the preparation of zirconia-silica mixed oxides. The zirconia-silica mixed oxide can be prepared even with deposition of ZrO_2_ on the silica surface either by precipitation or impregnation [225]. During the impregnation technique, the silica are suspended many times in aqueous solution of Zr(NO_3_)_4_ in the presence of ammonia [223,225].

At higher zirconia content, the opportunity for the crystallinity increases. The density of aerogels is also rising in the function of growing ZrO_2_ content and the porosity transforms from macroporous rather to microporous [223]. Intermediate ratios of Si/Zr give rise to mesoporous solids with a narrow pore size range. Both acid and base catalyzed sol-gel processes yield materials with similar surface areas, but with different physical properties. Acid catalyzed condensation leads to development of loose structures with fractal character and smaller particle sizes [224,227]. The mass fractal dimension extends, *i.e.,* the structure becomes more compact by increasing the molar ratio of H_2_O/metal alkoxide. The alkoxide precursors form aggregates of three-dimensional branched polymers in acidic medium [226]. The base catalyzed condensation produces compact branched cluster aggregates from larger particles and wide pore distributions [224,226]. The higher the zirconia content, the lower the surface area is (Table 1) [223,224,225]. The embedded zirconia reduces the size of the silica domains.

The highest density of Brønsted acid sites can be achieved on the samples of 20 mol % ZrO_2_, while the highest total acid site density at 75 mol % ZrO_2_ owing to the high Lewis site density [223]. The surface acidity in terms of numbers of Lewis and Brønsted sites proved to be independent of the type of catalyst applied in the sol-gel processing [224].

In order to improve the catalytic properties of ZrO_2_–SiO_2_ aerogels, the samples are modified with other metallic oxides. The substitution of Si^IV^–O- by Zr^IV^–O- bonds significantly affects the chemical properties of the isolated VO_4_ units in the silica matrix. The reducibility of the surface vanadium oxide species increases [229]. Introducing NiO into the aerogel matrix effectively increases the catalytic feature [228,230]. The Ni/ZrO_2_/SiO_2_ aerogels catalysts were synthesized via three different routes: impregnation of ZrO_2_–SiO_2_ aerogels with aqueous solution of Ni(NO_3_)_2_; impregnation of pure SiO_2_ aerogels with a mixed aqueous solution of Ni(NO_3_)_2_ and ZrO(NO_3_)_2_·2H_2_O; and sol-gel procedure from precursors Ni(NO_3_)_2_ / ZrO(NO_3_)_2_ · 2 H_2_O / Si(OC_2_H_5_)_4_ [228,230]. The sol-gel technique results in the best properties. The BET surface area of sol-gel derived samples is obviously higher (470 m^2^ g^-1^) than in aerogels synthesized by other routes (~280 m^2^ g^-1^); the pore size distribution is more uniform; the surface acidity and the density of Zr–O–Si bond are larger. However, the NiO content significantly reduces the surface area, from ~900 to 300–500 m^2^ g^-1^ [228]. The presence of ZrO_2_ also improves the interaction between Ni species and ZrO_2_-SiO_2_ supports [228].

Nanocomposites containing nickel oxide [231,232], copper oxide [232], chromium oxide [233], molybdenum oxide [234], or iron oxide [235,236,237,238,239] nanoparticles dispersed in the porous amorphous silica matrix are of interest due to their attractive magnetic, optic, electric, and catalytic properties. The large surface area of aerogels is expected to exhibit enhanced catalytic performances. Two types of synthesis have been reported for incorporation of *nickel* or *copper oxides* into the silica networks. In one of them, the wet silica gels prepared by polycondensation of TEOS and amino-substituted alkoxysilane mixture are impregnated with alcoholic solution of metal salts [232]. In a typical sol-gel route, the nickel or copper salts are subjected to react with amino-substituted alkoxysilane to obtain e.g., [(CH_3_O)_3_Si(CH_2_)_3_NHCH_2_CH_2_NH_2_]_n_M^2+^ complex. The hydrolysis and condensation rate of this complex can be well compared to that of TEOS coprecursor [232]. Using the sol-gel route with coprecursor yields larger porosity and surface areas. There is an example for the application of inorganic Ni salt ⌠Ni(NO_3_)_2_ ∙ 6H_2_O⌡ in the sol-gel processing [231]. The cogelation of Ni nitrate and TEOS precursors produces mainly mesoporous gel samples with very narrow pores (2.0–5.0 nm). The surface area is 600–900 m^2^ g^-1^, while the total pore volume is 0.5–2.0 cm^3^ g^-1^. A very little microporous volume can be detected in the NiO–SiO_2_ aerogel samples. The crystallite sizes of NiO ranges from around 10 nm to 2–3 nm depending on the supercritical drying conditions. The particle distribution over the silica matrix is very homogenous [231].

The sol-gel derived *chromium oxide*–silica aerogels with 5–30 mol % Cr content possess a large porosity (~ 700 m^2^ g^-1^ surface area) [233]. Chromium acetylacetonate and TMOS provide the precursor in an acidic medium. Chromium oxide oligomers, such (Cr–O–Cr–O–Cr)*_x_* exist on the surface of silica gel matrix, where the oxidation state of chromium ions are Cr^3^^+^ and Cr^6^^+^. Dichromate or polychromate species like Cr_2_O_7_^2-^ or Cr_3_O_10_^2-^ predominate on the surface of heat treated silica. Cr^6^^+^ species are anchored to the silica support via Si–O–Cr bonds [233]. The CrO*_x_* –SiO_2_ aerogels are amorphous up to 500 ºC, calcination at 500 ºC results in a crystallization of *α*-Cr_2_O_3_ phase. The *α*-Cr_2_O_3_ phase appears above 30 mol % chromium content.

*Fe_2_O_3_* –SiO_2_ mixed oxide composites with various compositions have been synthesized by the sol–gel method for applications in environmental protections, in catalysis and as novel magnetic materials. A typical sol-gel method produces iron oxide–silica alcogels from a small amount of iron(III) nitrate nonahydrate [Fe(NO_3_)_3_·9 H_2_O], TEOS or TMOS precursors, and gelation agents (nitrogen-containing bases) in ethanol [237]. Using an organic epoxide as gelation agents during the sol-gel processing, large iron oxide content can be achieved in the silica matrix [235,240]. By this novel method, several metal mixed oxides, including iron(III) oxide, have been prepared using common salts of Cr^3+^, Al^3+^, In^3+^, Ga^3+^, Sn^4+^, Hf^4+^, Zr^4+^, Nb^5+^, Ta^5+^, and W^6+^ and silica precursors [235,240]. The epoxide has a promoting role in the gelation. The simultaneous oxolation and olation of the [Fe(H_2_O)_6_]^3+^ ions and the silicon alkoxides yield Fe_2_O_3_ –SiO_2_ mixed oxide composites, in which two oxide components are well dispersed. Samples containing *α*-, *γ*-, and *ε*-Fe_2_O_3_–SiO_2_ composites can be simply obtained by controlling the temperature of calcination [235]. The aerogel nanocomposite materials prepared in the presence of an organic epoxide from FeCl_3_ · 6 H_2_O and TEOS or TMOS can be characterized with surface areas ranging from 350–450 m^2^ g^-1^; mesoporosity (pore diameters = 2–50 nm); and narrow pore size distributions. Surface area of 17 m^2^ g^-1^ has been detected in the pure Fe_2_O_3_ aerogel powders [236]. Applying ferric acetylacetonate and TMOS sol-gel precursors, the obtained aerogel composites possess really high porosity (760–870 m^2^ g^-1^). The influence of the rate of iron(III) and silicon oxides on the porosity is weak, not unambiguous (Table 1) [235,236]. Iron(III) oxide and silica dispersion throughout the bulk material produced in a sol-gel route is extremely uniform on the nanoscale [235]. Active catalysis requires appropriate iron dispersion on the silica surface, including a strong electronic interaction. The active catalytic properties of Fe_2_O_3_–SiO_2_ composites can be attributed to the Lewis acidity of iron oxide. Lewis-type sites can be associated with low-coordinated iron sites [237].

**Table 1:** Influence of metal ions on silicate structures.

*Aerogels*	*Precursors*	*Specific surface area* (m^2^ g^-1^)	*Average pore diameter* (nm)	*Reference*
**Li_2_O–SiO_2_**	Li silicate + TEOS	300	20–30	[156]
**MgO–SiO_2_**	Mg(NO_3_)_2_ + TEOS	200–340; 520	10–15	[162,164]
5% MgO	Mg(NO_3_)_2_ + TEOS	200	10	[162,164]
10% MgO		242	15	
**CaO–SiO_2_**	Ca(NO_3_)_2_ + TEOS	30–260	10–50	[167,168]
0% CaO	Ca(NO_3_)_2_ + TEOS	1000	~30	[167,168]
34% CaO		260	> 30	
44% CaO		53	~30	
66% CaO		33	< 10	
0% CaO	Ca(NO_3_)_2_ + TEOS	1280	~3	[133]
10% CaO	+ 20% PDMS	780	~3	
20% CaO		600	~11	
**Al_2_O_3_–SiO_2_**	AlX_3_ + TEOS	10–1050	5–15	[98]
50% Al_2_O_3_	Al(NO_3_)_3_ + TEOS	630	12	[98]
	Al(O*^i^*Pr)_3_ + TEOS	820	9	
	Al(OOCCH_3_)_2_(OH)	84	5	
	Aln + TEOS + PDMS	340	10	[98]
0% Al_2_O_3_	Boehmite sol + TEOS	740	66	[171]
5% Al_2_O_3_		800	65	
15% Al_2_O_3_		420	53	
25% Al_2_O_3_		270	50	
1% Al_2_O_3_	Al(*^s^*OBu)_3_ + TEOS + Ethyl acetoacetate	630–720	15–20	[172]
**TiO_2_–SiO_2_**	Ti(OR)_4_ + Si(OR)_4_	400–700	10–30	T1, T2
0% TiO_2_	Ti(O*^iso^*Pr)_4_ + TEOS	895	92	T1
5% TiO_2_		685	136	
10% TiO_2_		620	152	
20% TiO_2_		400	176	
	Ti(O*^i^*Pr)_4_ + TEOS + TMCS	540–640	40–110	T1
0% TiO_2_	Ti(O*^iso^*Pr)_4_ + TMOS	750	6–30	T6
10% TiO_2_		1080	11	
20% TiO_2_		896	13	
50% TiO_2_		700	11	
**V_2_O_5_–SiO_2_**		200–1000	10–100	
10% V_2_O_5_	VOTIP / V(acac)_3_ + TEOS	840	9	[208,209]
20% V_2_O_5_		600	13	
30% V_2_O_5_		430	7	
0% V_2_O_5_	V(acac)_3_ + TEOS	1070	93	[216]
5% V_2_O_5_		860	60	
10% V_2_O_5_		260	42	
20% V_2_O_5_		680	50	
**ZrO_2_–SiO_2_**	Zr(OPr)_4_ + TEOS	100–500	10–100	[220,221,222]
9% ZrO_2_	Zr(OPr)_4_ + TEOS	460		[220]
20% ZrO_2_	Zr(OPr)_4_ + TMOS	300		
50% ZrO_2_		220		[221,222]
75% ZrO_2_		150		
Impregnated		93		
Precipated		98		
**Fe_2_O_3_–SiO_2_**	Fe(NO_3_)_3_ + TMOS	400–600	20–30	[234]
83% Fe	FeCl_3_ + TEOS	400	29	[232]
67% Fe		430	28	
50% Fe		390	18	
**NiO–SiO_2_**	Ni nitrate + TEOS	600–900	2–5	[228]

## 4. Conclusions

The potential applications of silica and various silicate aerogels depend mostly on their nanostructure, thus the study of the porous structures is of primary importance. Numerous investigations have proved that the structure of porous materials can be appreciably tailored by variation of synthesis conditions. The objectives of this review are to summarize and elucidate the effects of chemical conditions on the nanoporous structure of sol-gel derived pure silica and mixed oxide–silica aerogels. The most important factor for the modification of structures is the relative rate of the hydrolysis and condensation reactions. The gelation processes can be flexibly regulated by the solution parameters, the kinetics, and mechanism of sol–gel technique. The review presents the influences of starting materials; pH; catalysis; precursor concentration; water and organic solvent content on the nanostructure of silica-containing aerogels. Only a brief description of chemical agent effects (organic entities; surfactants; polymers, and templates) is provided in this paper. Since the evaluation of the effects of embedded metal ions has been less emphasized in the reported monographies, greater attention has been paid to the effect of metal ions inserted in the silica matrix.
